# Micro-MRI improves the accuracy of clinical diagnosis in cerebral small vessel disease

**DOI:** 10.1093/braincomms/fcab070

**Published:** 2021-04-08

**Authors:** Hidehiro Ishikawa, Atsushi Niwa, Shinya Kato, Yuichiro Ii, Akihiro Shindo, Keita Matsuura, Yamato Nishiguchi, Asako Tamura, Akira Taniguchi, Masayuki Maeda, Yoshio Hashizume, Hidekazu Tomimoto

**Affiliations:** 1 Department of Neurology, Mie University Graduate School of Medicine, 2-174 Edobashi, Tsu, Mie 514-8507, Japan; 2 Radioisotope Facilities for Medical Science, Advanced Science Research Promotion Center, Mie University, Tsu, Mie, 514-8507, Japan; 3 Department of Neuroradiology, Mie University Graduate School of Medicine, 2-174 Edobashi, Tsu, Mie 514-8507, Japan; 4 Department of Neuropathology, Fukushimura Hospital, Aichi 441-8124, Japan

**Keywords:** micro-MRI, small vessel disease, cerebral microbleeds, cerebral superficial siderosis, cerebral amyloid angiopathy

## Abstract

Even with postmortem pathological examination, only limited information is provided of the foci of *in vivo* clinical information. Cerebral small vessel disease, which is associated with ageing, dementia and stroke, highlights the difficulty in arriving at a definitive diagnosis of the lesions detected on *in vivo* radiological examination. We performed a radiological−pathological comparative study using *ex vivo* MRI to examine small cerebral lesions. Four patients with small vessel disease lesions detected on *in vivo* MRI were studied. Exact pathological findings of *in vivo* MRI-detected lesions were revealed. The ischaemic lesion after 17 days from onset showed positivity for peroxiredoxin, cluster of differentiation 204 and glial fibrillary acidic protein, indicating sterile inflammation and neuroprotective reaction. Cortical microinfarcts beneath the cortical superficial siderosis were associated with inflammation from the superficial layer in a patient with cerebral amyloid angiopathy; in this patient, a bilinear track-like appearance of the cortical superficial siderosis on the *ex vivo* MRI was compatible with iron deposition on the pia matter and within cortical layers II–III. An *in vivo* MRI-detected cerebral microbleed was revealed to be heterogeneous. An *in vivo* MRI-detected cerebral microbleed was revealed to be a venous angioma. Furthermore, a neuropathologically confirmed embolic cerebral microbleed was firstly detected using this method. Our results suggest that *in vivo* MRI-detected lobar cerebral microbleeds can be caused by non-cerebral amyloid angiopathy aetiologies, such as microembolism and venous angioma. Venous angioma and embolic microbleeds may mimic cerebral amyloid angiopathy markers on *in vivo* MRI. To clarify the clinical importance of these lesions, we should investigate their rate and frequency in a large cohort of healthy individuals and patients with cardiac risk factors. Thus, we provide evidence that *ex vivo* micro-MRI improves the clinical diagnosis of small vessel diseases.

## Teaser

This study introduces unique method for radiological**−**pathological comparative research using micro-MRI. The micro-MRI was used as bridge to assess findings of *in vivo* MRI and neuropathology. Novel neuropathological findings of *in vivo* MRI-detected small vessel disease, such as cerebral microbleeds, cortical microinfarcts, and cortical superficial siderosis were described.

## Graphical Abstract

**Graphical Abstract:**
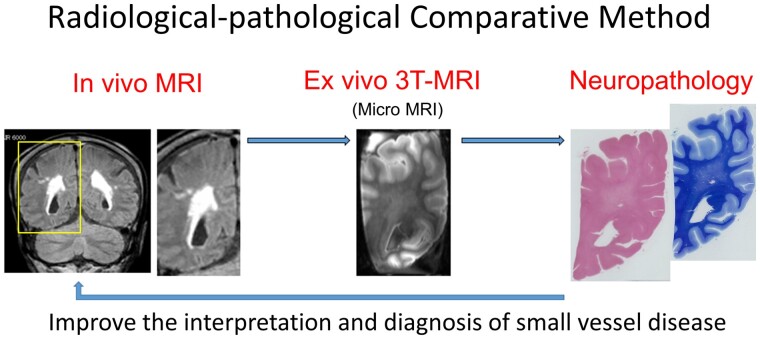

## Introduction

In many neurological diseases, an autopsy is required for definite diagnosis. However, only limited sections of autopsied tissue can be analysed through histopathological examinations. Cerebral small vessel disease is a representative example of the difficulty in making a definitive diagnosis of the exact lesions detected by *in vivo* radiological examination. Clinicopathological and clinicoradiological studies have indicated that treatment and prevention for small vessel disease would be beneficial to decelerate cognitive decline.^1–5^ For early intervention in small vessel disease, improvement of the disease’s clinical diagnosis is crucial. 

Lobar cerebral microbleeds (CMBs), cortical microinfarcts (CMIs), cortical superficial siderosis (cSS) and white matter hyperintensity are key MRI markers for cerebral amyloid angiopathy, which is a form of small vessel disease.^1^^,^^6^ These *in vivo* MRI findings are not always caused by cerebral amyloid angiopathy, and the exact underlying pathology remains unclear. To solve this problem, it is important to investigate small vessel disease using *in vivo* MRI.^7^^,^^8^

In most radiological–pathological comparative studies, MRI is used to examine *in vivo* human tissues.^9^ In the clinical setting, since the MRI machines are dedicated to patient use during the day, the time in which *ex vivo* brains can be scanned is restricted to after hours (night time).^10^^,^^11^ In addition, it is often difficult to obtain thin brain slices (thinner than 10 μm) that include *in vivo* MRI-detected small vessel disease for pathological examination. Therefore, the exact pathologies of *in vivo* MRI-detected small vessel disease lesions have been seldom reported.^12^ Micro-MRI has been designed for experimental purposes, the performance time is short and because of the microcoils, its spatial resolution is much superior to that of regular 3-T MRI for the whole brain. Few micro-MRI studies have examined human tissue.^13–15^*In vivo* MRI-detected small vessel disease can be efficiently assessed by neuropathological examinations using micro-MRI as a ‘bridge’. In this study, we describe an efficient method of *in vivo* radiological−*ex vivo* pathological comparison and novel comparative findings of small vessel disease in patients with *in vivo* MRI-defined small vessel disease.

## Materials and methods

### Subjects

We selected patients who had available *in vivo* MRI scans and findings of small vessel disease detected using *in vivo* MRI. Only patients whose nearest family provided informed consent allowing an autopsy for diagnostic and scientific purposes were included in this study. Four patients with *in vivo* MRI-defined small vessel disease were subjected to a radiological**−**pathological study (see details in Supplementary material and Supplementary Figs 1–3). This study was approved by the Ethics Committee of Mie University Hospital (approval number 2498) and conformed to the tenets of the Declaration of Helsinki.

### *In vivo* 3-T MRI protocol

*In vivo* 3-T MRI was performed using a 3-T MR scanner (Ingenia, Philips Health Care, Best, the Netherlands), using a 32-channel phased-array head coil. Diagnostic imaging included 3D fluid attenuated inversion recovery (FLAIR), 3D T_1_-weighted imaging (WI), diffusion WI and susceptibility WI (SWI). 3D FLAIR parameters included a 250 mm field of view (FOV) with a 256 × 184 matrix, a section thickness of 1 mm with a 0.57 mm overlap, no parallel imaging, a repetition time (TR)/echo time (TE) ratio of 6000/378 (shortest), and an inversion time (TI) of 2000 ms; the number of signals acquired was two and the acquisition time was 4 min 42 s. SWI parameters included a FOV of 230 mm with a 320 × 251 matrix, a section thickness of 0.8 mm over contiguous slices, a minIP of 5 mm, a TR/TE ratio of 22/11.5 (in-phase), 37 (shifted); number of signals acquired, 1; flip angle 20°; and an acquisition time of 6 min 42 s. 3D T1WI parameters included a FOV of 260 mm; matrix, 288 × 288; section thickness, 0.9 mm; TR (ms)/TE (ms) ratio, 7.6 (shortest); TE (shortest), 3.6 ms; flip angle, 10°; and an acquisition time of 4 min 42 s.

### *Ex vivo* MRI protocol

Micro-MRI scanning was performed using a 3-T MR scanner (LabPET/MRI 3016, TriFoil Imaging, Inc., Northridge, CA, USA), using a rat body coil. The formalin-fixed brain was cut to fit the plastic box for setting in the coil in advance. The brain block was placed in the plastic box filled with salt-free water at 35°C. We employed an MR protocol with T1WI, T2WI, T2*WI and FLAIR. Details of the T2WI protocol are as follows: FOV, 80 × 80 mm; slice thickness/gap, 1.0/0.5 mm; acquisition matrix, 256 × 256; pixel size, 0.3125 × 0.3125 mm; TR, 4800 ms; TE, 68 ms; flip angle, 90°; number of averages, 2 and acquisition duration, 310 s. Details of the T2*WI protocol are as follows: FOV, 80 × 80 mm; slice thickness/gap, 1.0/0.5 mm; acquisition matrix, 256 × 256; pixel size, 0.3125 × 0.3125 mm; TR, 400 ms; TE, 16 ms; flip angle, 50°; number of averages, 4 and acquisition duration, 482 s. Details of the FLAIR protocol are as follows: FOV, 80 × 80 mm; slice thickness/gap, 1.0/0.5 mm; acquisition matrix, 264 × 128; pixel size, 0.625 × 0.15625 mm; TR, 7000 ms; TE, 68 ms; TI, 2000 ms; flip angle, 90°; number of averages, 2 and acquisition duration, 423 s.

### Neuropathology

This study used formalin-fixed brain tissues from four patients with small vessel disease detected using *in vivo* MRI. After postmortem *ex vivo* micro-MRI scanning, brain blocks were dehydrated and embedded in paraffin. Brain blocks were cut in 8 μm thick serial sections using a microtome. Standard histologic staining was performed on these serial sections, which included haematoxylin/eosin staining and Kluver–Barrera staining. Further immunohistochemistry was performed to clarify the aetiology of small vessel disease (Supplementary Table 1).

### Radiological−pathological comparative study procedure

**Figure 1:**
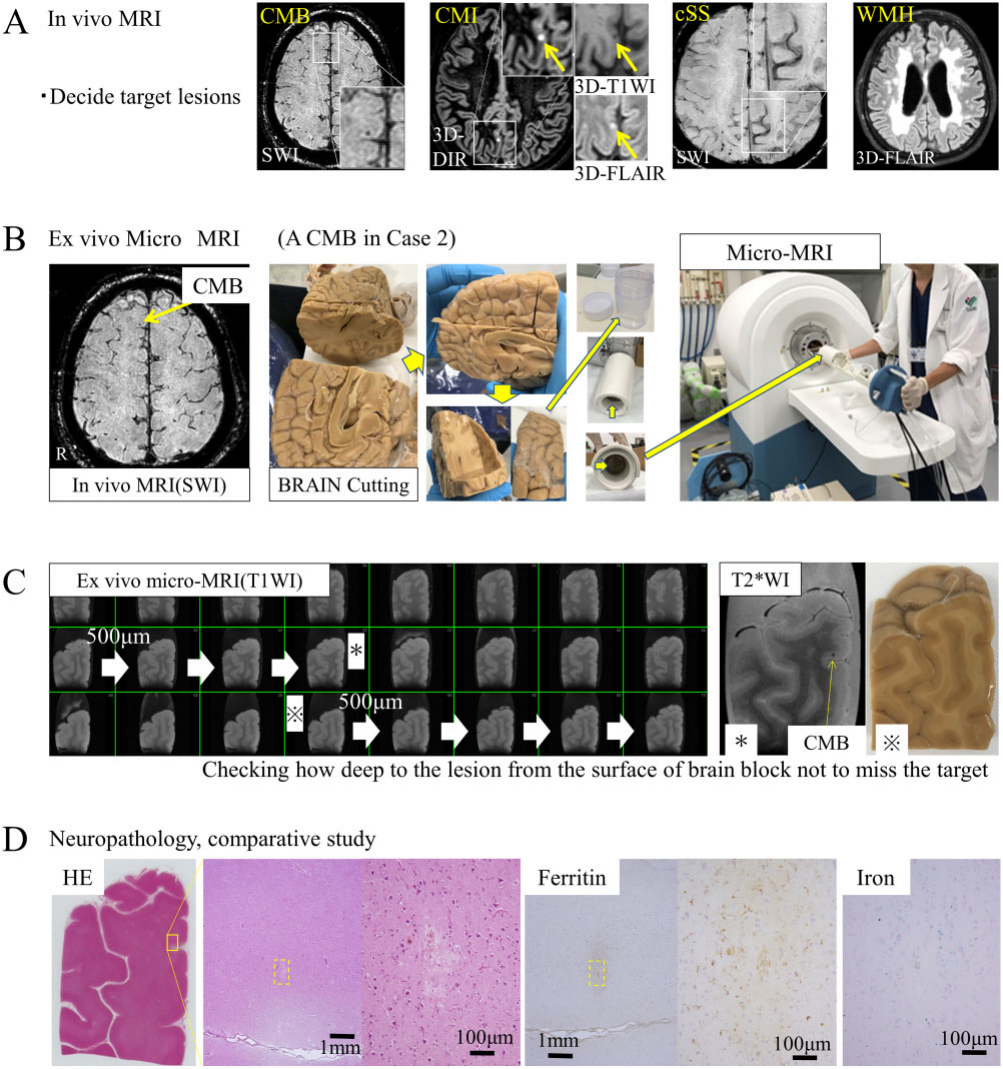
**Radiological−pathological comparative method for small vessel disease.** First, the target lesion should be retrospectively detected on *in vivo* MRI (**A**). Second, a brain block made to fit the field of view (80 × 80 mm) is set in the micro-MRI (**B**). Third, the brain block is sliced for neuropathological analysis while carefully inspecting the depth of the lesion from the surface of the brain block using *ex vivo* MRI (**C**). Finally, the brain sample is dehydrated, embedded in paraffin and cut in 8 μm-thick serial sections on a microtome at the estimated depth determined by *ex vivo* MRI. The lesion can be observed on glass slides for standard haematoxylin and eosin staining and additional immunohistochemistry (**D**).

The radiological**−**pathological comparative method for small vessel disease is summarized in Fig. 1. Neuroimaging markers of SVD on *in vivo* MRI were defined according to the STRIVE consensus criteria.^16^ Formalin fixed brains were cut in blocks that included the target lesion and were of a size equivalent to the FOV used in the *ex vivo* micro-MRI examination.

### Data availability statement

The data that support the findings of this study are available from the corresponding author upon reasonable request.

## Results

**Figure 2:**
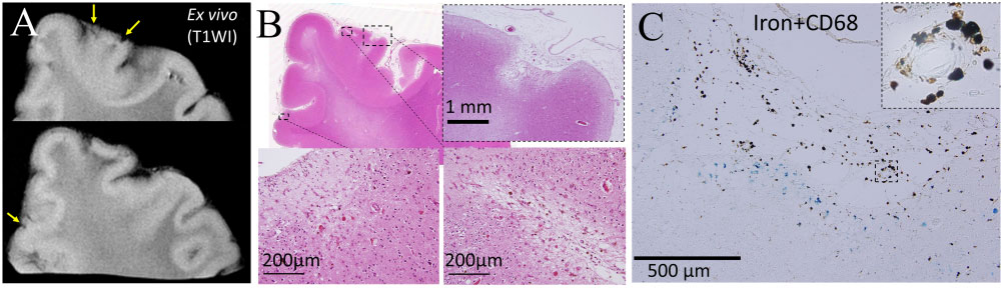
**Cortical microinfarcts revealed by the radiological−pathological comparative method with micro-MRI.** CMIs 0.7−5 mm were detectable as low intensity on the T_1_-weighted image of 3-T micro-MRI (**A**). The sizes and shapes on micro-MRI were almost the same using haematoxylin and eosin staining of the neuropathology (**B**). CMIs in Case 1 showed iron positivity in the cortical area (**C**). Some CD68-positive cells showed positivity for iron on double staining of iron and CD68, compatible with iron containing macrophages (inset of **C**). CD, cluster of differentiation; CMI, cortical microinfarct.

**Figure 3:**
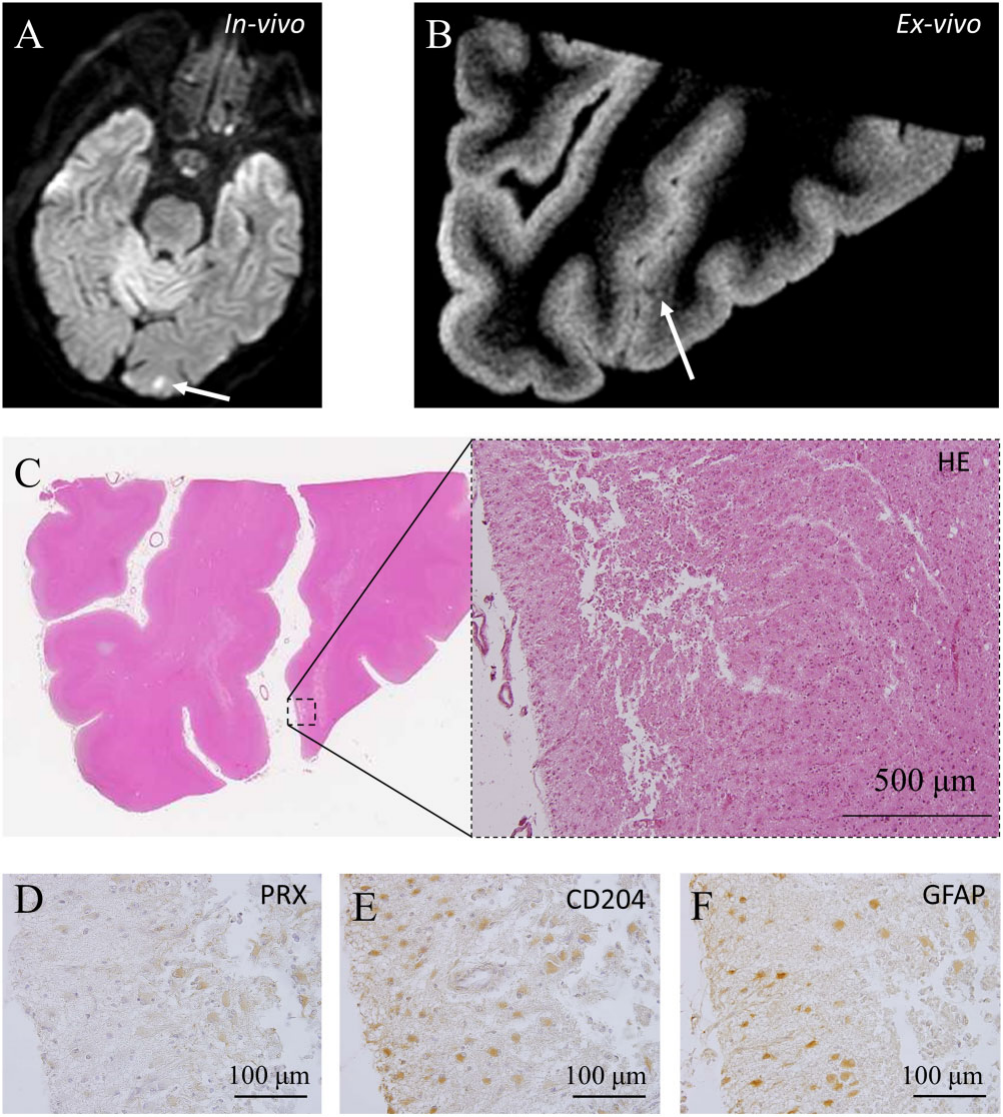
**An acute cortical microembolism in Case 4.** An acute cortical microembolism was observed on *in vivo* MRI (**A**). A hypointense lesion was observed on the T_1_-weighted image of *ex vivo* micro-MRI (**B**). The radiologically suspected ischaemic lesion was neuropathologically shown to be a microinfarction that extended to the subcortical area (**C**). The microinfarction was positive for peroxiredoxin (**D**), CD 204 (**E**) and GFAP (**F**), and suggestive of inflammation and a neuroprotective reaction. CD, cluster of differentiation; GFAP, glial fibrillary acidic protein.

**Figure 4:**
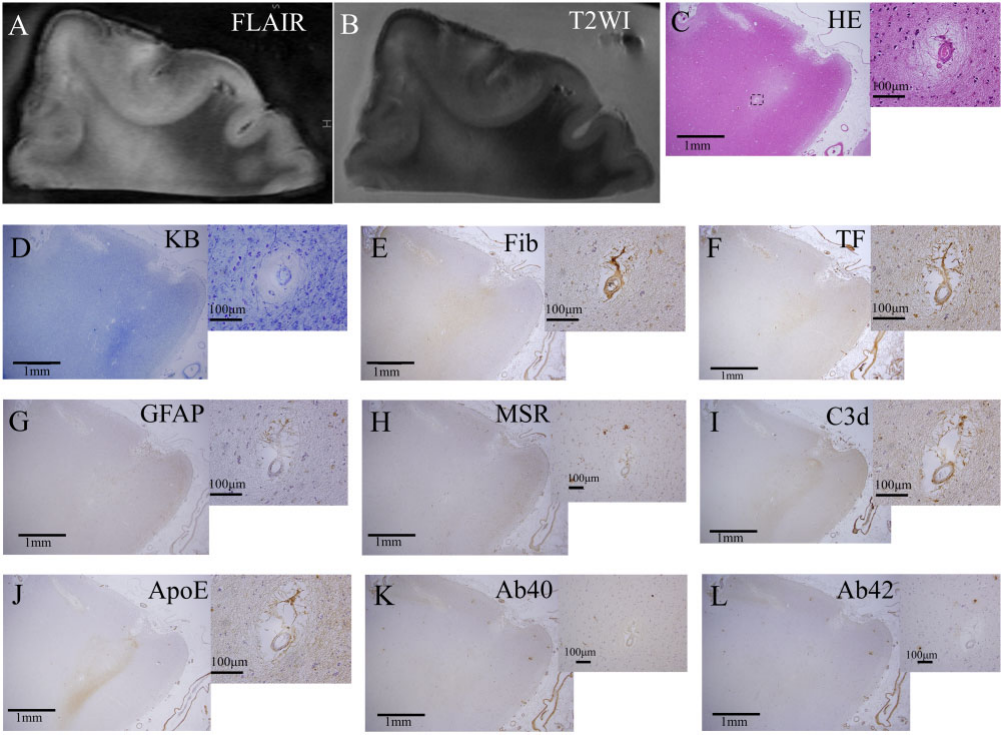
**Polioaraiosis and a white matter lesion revealed by the radiological−pathological comparative method with micro-MRI.** Cortical hyperintensity on FLAIR (**A**) and T2WI (**B**). Cortical hyperintensity is shown by the pale area on HE stained sections (**C**). Some enlarged perivascular spaces were observed (inset of each panel). The hyperintense area included the cortex, as observed by (KB) staining (**D**). Fib (**E**) and TF (**F**) were positive, including in vessel walls, suggesting a linkage with the damaged blood−brain barrier. This area had mild positivity for GFAP, suggestive of the presence of astrocytes (**G**). Although MSR was negative in the hyperintense area (**H**), some MSR-positive cells were observed around the vessels with an enlarged perivascular space (inset of **H**). C3d (**I**) and ApoE (**J**) were positive in the hyperintense area. Aβ40 was negative in the vessels with enlarged perivascular space, suggesting that Aβ clearance was maintained in such vessels (inset of **K**). Aβ42-positive plaques were also positive for MSR-positive cells, suggesting that the macrophages captured Aβ42 (inset of **H** and **L**). Aβ, amyloid beta; ApoE, apolipoprotein E; Fib, fibrinogen; FLAIR, fluid attenuated inversion recovery; GFAP, glial fibrillary acidic protein; HE, haematoxylin and eosin; KB, Klüver−Barrel; MSR, macrophage scavenger receptor; T2WI, T_2_-weighted imaging, TF; transferrin.

**Figure 5:**
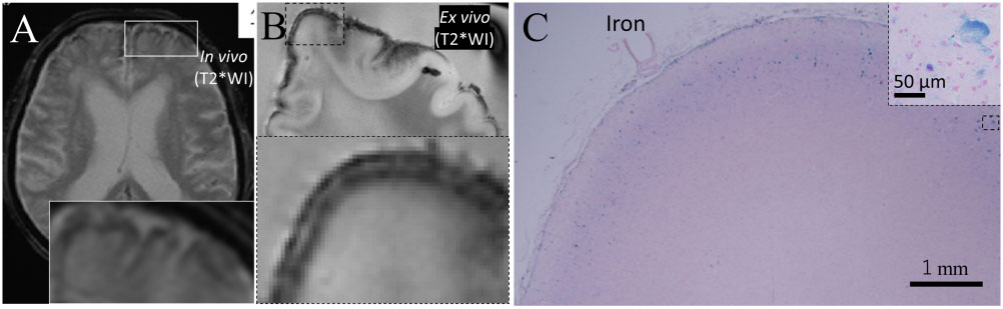
**Comparative findings of cortical superficial siderosis.** cSS in Case 1 was detected as a hypointense cortical rim on T2*WI of *in vivo* MRI (**A**). cSS was detected as curvilinear hypointensity following the cortical surface on *ex vivo* micro-MRI that was consistent with the *in vivo* images (**B**). The low-intensity area was consistent with the iron-positive area on the iron staining of neuropathology (**C**). Iron-positive anuclear foamy structure was observed in cortical layers II–III (inset of **C**).

*In vivo* MRI-detected small vessel disease findings from four patients were examined (Table 1). Case 1 was neuropathologically diagnosed with cerebral amyloid angiopathy, while the others were diagnosed with non-cerebral amyloid angiopathy. Microinfarcts were detected on micro-MRI in Cases 1, 2 and 4. Three Tesla *ex vivo* micro-MRI was able to detect CMIs, including those <1 mm (Fig. 2A). Inflammatory cells propagated from the superficial to the deep layers along the perivascular area in the patient with cerebral amyloid angiopathy (Fig. 2B). The comparative study revealed cells positive for iron and cluster of differentiation (CD) 68 around the CMIs (Fig. 2C). An acute CMI in Case 4 was revealed to be due to microembolism showing positivity for peroxiredoxin, CD204, and glial fibrillary acidic protein (GFAP) (Fig. 3). Cortical hyperintensity on FLAIR and T2WI in Case 1 corresponded to positivity for serum protein exudate, astrocytic markers and the complement component C3d (Fig. 4). cSS was detected using *in vivo* and *ex vivo* MRI in Case 1 (Fig. 5A and B). A bilinear track-like appearance in the *ex vivo* MRI of Case 1 was compatible with iron deposition on the cortical surface and within the cortical layer (Fig. 5B and C). These were mostly cells of monocyte/macrophage lineage, but some were iron-positive foamy structures (Fig. 5C) negative for Aβ, GFAP and CD68 but slightly positive for ubiquitin (Supplementary Fig. 4). Although T2* low intensity, suggestive of cSS, was clearly revealed on the *ex vivo* MRI of Case 2, it was not detectable on *in vivo* MRI, and there were no cerebral amyloid angiopathy findings on the neuropathological examination (Supplementary Fig. 5A–C). In Case 2, iron-positive foamy structures were not observed (Supplementary Fig. 5D), and neither were cerebral amyloid angiopathy findings (Supplementary Fig. 5E).

**Table 1:** Summary of four cases with *in vivo* MRI-defined small vessel disease

Cases	1	2	3	4
Neuropathological diagnosis	Alzheimer’s disease, cerebral amyloid angiopathy	Amyotrophic lateral sclerosis	Corticobasal degeneration	Stroke
Age (years) at death	81	77	62	76
Sex (M: Male/F: Female)	M	M	M	M
Hypertension	+	+	−	−
Hyperlipidemia	−	−	−	−
Diabetes mellitus	−	+	−	−
Smoking	−	+	+	+
Atrial fibrillation	−	+	–	+
*In vivo* MRI	1.5-T	3-T	3-T	1.5-T
Cortical microinfarcts	−	−	−	+
Cerebral microbleeds	+	+	+	−
Cortical superficial siderosis	+	−	−	−
*Ex vivo* MRI	3-T	3-T	3-T	3-T
Cortical microinfarcts	+	+	−	+
Cerebral microbleeds	–	+	+	−
Cortical superficial siderosis	+	+	−	−
Neuropathology				
Cortical microinfarcts	+	+	−	+
Cerebral microbleeds	+	+	−	−
Cortical superficial siderosis	+	−	−	−
Duration between *in vivo* MRI and *ex vivo* MRI/neuropathology	9 months	46 days	1.5 years	17 days

**Figure 6:**
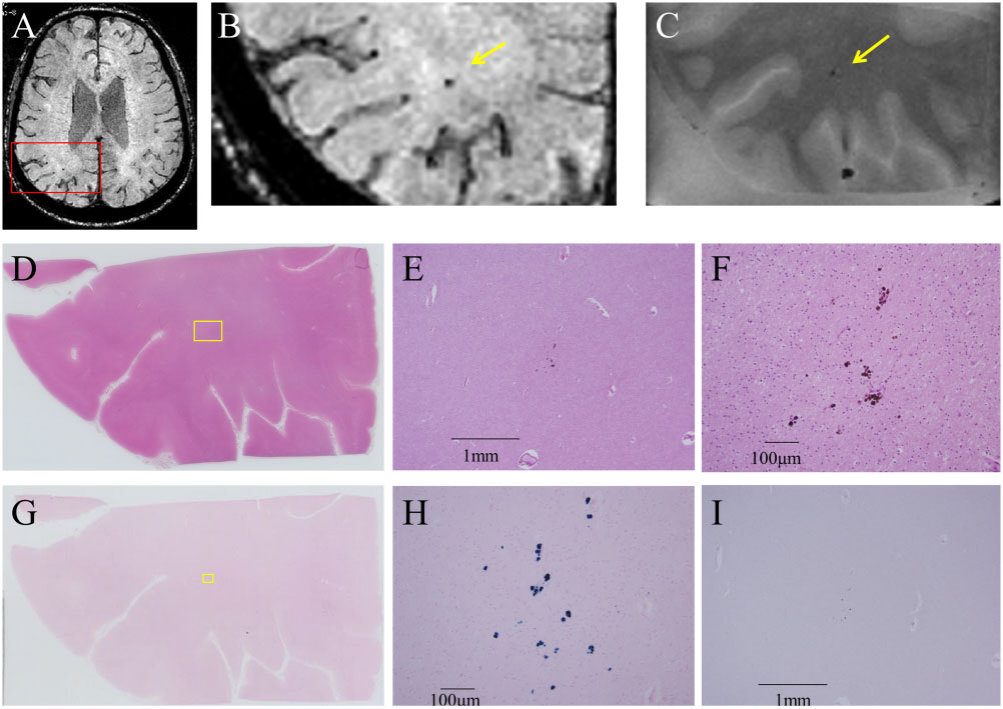
**Comparative findings of a cerebral microbleed in Case 2.** A cerebral microbleed (CMB) was observed as hypointensity on the susceptibility weighted image (SWI) of *in vivo* MRI (**A**). The hypointensity on SWI of the *in vivo* MRI was also detected as hypointensity on the T_2_*-weighted image of *ex vivo* micro-MRI (**B** and **C**). Neuropathological study was performed to match the exact location of the lesion (**D**). Hemosiderin deposits were observed in the lesion by haematoxylin and eosin staining (**E** and **F**). These hemosiderin deposits were positive for iron staining (**G** and **H**). There were no Aβ-positive vessels detected by Aβ staining (**I**).

**Figure 7:**
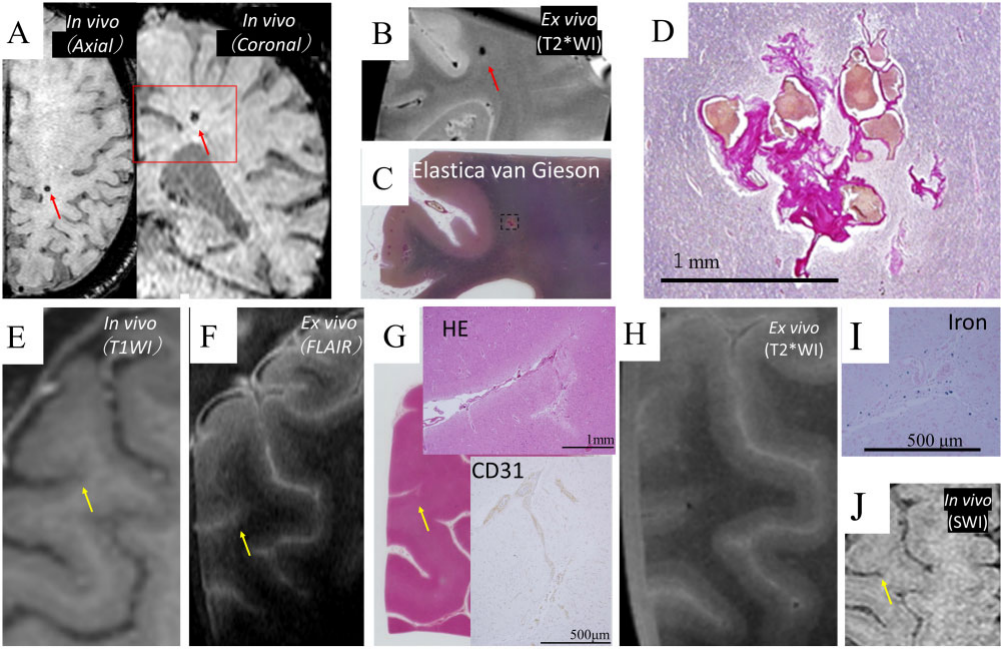
**Venous angioma clinically diagnosed as a cerebral microbleed on *in vivo* MRI, and embolic microbleed revealed by the radiological−pathological comparative method with micro-MRI.** A lesion in Case 3 diagnosed as CMB on SWI of *in vivo* MRI (**A**) was detected as a hypointensity lesion on T2*WI of *ex vivo* MRI (**B**), which was compatible with CMB. The lesion was revealed to be a non-CMB by the Elastica van Gieson staining of neuropathology (**C**). The lesion had vessel walls without internal elastic lamina, which indicated venous angioma (**D**). Case 2 had a low-intensity lesion on T1WI of *in vivo* MRI that constituted an ambiguous finding of CMI (**E**). The lesion showed high intensity on FLAIR of *ex vivo* MRI (**F**) and was confirmed as a CMI due to microembolism using neuropathological analysis, which showed fibrinoid degeneration in the adjacent CD31-positive pial arteriole meandering in the CMI (**G**). The CMI was identified as hypointense on *ex vivo* micro-MRI (**H**). Iron staining was positive on the slide showing meandering vessels in CMI (**I**). The site showed hypointensity on retrospective assessment on SWI of *in vivo* MRI (**J**). CD, cluster of differentiation; CMB, cerebral microbleed; CMI, cortical microinfarct; cSS, cortical superficial siderosis; FLAIR, fluid attenuated inversion recovery; SWI, susceptibility weighted image; T1WI, T_1_-weighted imaging; T2*WI; T_2_*-weighted imaging.

A comparative study of CMBs in the *in vivo* and *ex vivo* MRIs revealed hemosiderin deposits in the neuropathological specimens of Case 2 (Fig. 6). A lesion that had been radiologically diagnosed as CMB in Case 3 was revealed to be venous angioma on the neuropathological examination (Fig. 7A–D). An iron positive area was detected around the angioma (Supplementary Fig. 6). One lesion in Case 2 was an ambiguous CMI finding on *in vivo* MRI (Fig. 7E); this was recognized as a CMI using FLAIR in the *ex vivo* MRI (Fig. 7F) and continued to a patent microvessel that was occluded by microembolism on the neuropathological examination (Fig. 7G). The lesion was of low intensity, as revealed on T2*WI of the *ex vivo* MRI (Fig. 7H). Iron staining in the same location revealed iron deposits (Fig. 7I), indicating that the lesion was an embolic microbleed. The low intensity was retrospectively confirmed on *in vivo* MRI (Fig. 7J).

## Discussion

To date, *in vivo* MRI−*ex vivo* neuropathological studies have rarely been reported because of associated methodological complications and difficulties. In this study, we improved upon an existing method to shed new light on the field of small vessel disease.^10^^,^^11^ The major findings of the present study are as follows. First, we showed that the *in vivo* MRI−*ex vivo* pathological comparative method using micro-MRI was efficient. Second, findings of *in vivo* MRI, *ex vivo* MRI and neuropathology of cSS and CMIs in a patient with cerebral amyloid angiopathy indicated that there is a close spatial relationship between these pathologies. Third, this study revealed the heterogeneity of the neuropathology of CMBs, which includes venous angioma and embolic CMB.

Several radiological**−**pathological comparative studies using MRI for human brain tissue have been reported.^11^^,^^17^ It is often difficult to ensure correspondence between thin brain−specimen slices and small lesions. Our method using *ex vivo* micro-MRI can form a ‘bridge’ between *in vivo* MRI and histopathological findings. Postmortem changes can be excluded and additional micro-MRI scans can be performed repeatedly within 30 min per sequence at any angle if necessary to adjust the slice to correspond precisely to the *in vivo* imaging. The strongest advantage of this method is the size of the scanned images, which precisely corresponds to the neuropathological specimens and therefore allows for accurate comparisons. A study with similar protocol using a microcoil for *exvivo* MRI-pathological comparison has been reported.^15^ The previous study and our study support the advantage of using microcoils for accurate comparison between *exvivo* MRI and neuropathology. Furthermore, our study has improved the accuracy of comparison between *in vivo* MRI and *ex vivo* MRI images because of the shorter time protocol of *ex vivo* MRI than in the previous studies of overnight protocol.^10^^,^^12^^,^^15^

A very limited number of facilities with 7-T MRI have reported pathological comparisons with autopsy brain MRIs.^15^^,^^17^^,^^18^ Although 7-T MRI can provide clear images, it is not widely used in clinical practice. We confirmed that 3-T micro-MRI was able to detect lesions that were even smaller than 1 mm in size and have so far remained invisible in *in vivo* and *ex vivo* 3-T MRI.^3^^,^^11^ This high resolution was attributed to the microcoils. The smaller the FOV, the higher the spatial resolution. In addition to the time and resolution, another advantage of using micro-MRI is the easy management of temperature. Our previous study using *ex vivo* 3-T MRI revealed the importance of retaining a proper temperature to enhance the signal-to-noise ratio. Micro-MRI is equipped with a warming device to maintain a constant temperature.

*In vivo* MRI-detected small vessel disease markers of cerebral amyloid angiopathy are not always caused by cerebral amyloid angiopathy.^6^^,^^19^ We previously reported the differentiation of CMIs between cerebral amyloid angiopathy and micro-embolisms using *in vivo* 3-T MRI.^20^ In this study, CMIs in Case 1 were associated with cerebral amyloid angiopathy, while an acute (subacute at autopsy) CMI in Case 4 was consistent with the features of microembolism. The ischaemic lesion after 17 days from onset showed positivity for peroxiredoxin, CD204 and GFAP, indicating sterile inflammation and a neuroprotective reaction.^21^ The neuroprotective reaction may be related to being obscure of small ischaemic lesions on MRI images over time.

Neuroimaging markers for SVD on *in vivo* MRI in Case 1 have included white matter hyperintensity, above which there were CMIs in the cerebral cortex. Myelin loss, astrogliosis, and enlarged perivascular spaces were observed, and these findings were in line with neuropathological changes, which have been described in white matter hyperintensities.^22^ Furthermore, the pallor area in the white matter extended to cerebral cortex, consistent with the finding previously described as polioaraiosis.^23^ The neuropathological finding in Case 1 can be regarded precisely as tissue changes of hyperintensity detected on *in vivo* MRI.

Our data revealed that cSS has a close spatial relation to superficial CMIs. These findings indicate that the pathogenesis of CMIs may be related to inflammatory cells propagating along the perivascular area rather than to vascular occlusion. This result was in line with those of a recent cSS study.^24^ However, some unique findings were revealed in our patient. A bilinear track-like appearance of the cSS on the *ex vivo* MRI was compatible with iron deposition on the pia matter and within cortical layers II–III. While iron-positive foamy structures were observed in the iron-deposited cortical area in the patient with cerebral amyloid angiopathy, these structures were not observed in the iron-deposited cortical area in a patient with non-cerebral amyloid angiopathy. More data are needed to clarify whether the iron-positive structures are associated with cerebral amyloid angiopathy as well as the elapsed time course of iron deposition.

One lesion clinically diagnosed as CMB on *in vivo* MRI was a venous angioma. Iron deposits around the angioma appeared to have low intensity on the SWI of *in vivo* MRI. CMBs identified using *in vivo* imaging should be carefully interpreted because lobar CMBs may not be necessarily related to cerebral amyloid angiopathy. The fact that some of the *in vivo* MRI-defined lobar CMBs might not be real CMBs is an important consideration during the diagnosis of cerebral amyloid angiopathy.^25^ We previously reported that microembolisms can transform into microbleeds on MRI.^26^^,^^27^ A CMI due to microembolism in Case 2 showed iron deposits, which were captured as a CMB on both *ex vivo* and *in vivo* MRI. The microembolism was probably associated with atrial fibrillation. To the best of our knowledge, this is the first report of embolic CMB confirmed neuropathologically. There is evidence to suggest that lobar CMBs may be more common than deep CMBs in patients with atrial fibrillation.^28^^,^^29^ Atrial fibrillation is a common cause of cardioembolism. Considering these reports and our results, we should investigate the rate of embolic microbleeds in lobar areas, which potentially mimic cerebral amyloid angiopathy markers in both patients with cardiac risk factors and the healthy population.

### Limitations

Limitations of this study include the small sample size and the variation in the time between the MRI imaging and pathological examination. Our data were not enough to examine the validity of recent findings of clinico-radiological SVD study.^20^^,^^30^^,^^31^ One problem with studies on autopsy tissues is that it is often difficult to obtain family consent owing to cultural and religious reasons. In the future, it will be necessary to determine, using a large cohort, how the time from *in vivo* MRI to postmortem MRI and pathological examination influences these results.

## Conclusion

This method improves the diagnosis of cerebral small vessel disease because it allows for bidirectional assessment leading from i*n vivo* images to pathology or from pathological examination to interpretation of *in vivo* images. Further studies are required to establish the efficiency of our radiological**−**pathological comparative method and to clarify the exact pathology of *in vivo* MRI-detected small vessel disease. The next step using our methods would be clarification of the proportion of apparent CMBs due to other aetiologies diagnosed on in vivo MRI, and clinico-patholocical correlation of white matter hyperintensities. To clarify the uncertainty of such SVD lesions could improve the diagnosis of SVDs and enable to make accurate expectation for severity, progression and impairment.

## Supplementary Material

fcab070_Supplementary_Data

## Abbreviations

CD =: cluster of differentiation
CMB =: cerebral microbleed
CMI =: cortical microinfarct
CSS =: cortical superficial siderosis
FLAIR =: fluid attenuated inversion recovery
FOV =: field of view
GFAP =: glial fibrillary acidic protein
TE =: Echo time
TI =: inversion time
TR =: repetition time
WI =: weighted imaging
