# Supplementary material for: Micro-MRI improves the accuracy of clinical diagnosis in cerebral small vessel disease
Source: Brain Commun. 2021 Apr 8;3(2):fcab070. doi: 10.1093/braincomms/fcab070 (PMC8111066; doi:10.1093/braincomms/fcab070)
Supplement: fcab070_Supplementary_Data [file fcab070_supplementary_data.pdf]

Supplementary Material

This Supplemental Data has been provided by the authors to give additional information about their work.

SUPPLEMENTAL DATA

TABLE OF CONTENTS

List of Investigators
Supplementary Methods
    Case presentation
Supplementary Figure 1. Neuroradiology for Case 1
Supplementary Figure 2. Neuropathology for Case 1
Supplementary Figure 3. Neuroradiology for Case 2
Supplementary Figure 4. Immunohistochemistry of foamy structures in Case 1
Supplementary Figure 5. Comparative findings of superficial siderosis in Case 2
Supplementary Figure 6. Pathological findings of venous angioma in Case 3
Supplementary Table 1. Antibodies used in Neuropathology
Supplementary references

Micro-MRI improves the accuracy of clinical diagnosis in cerebral small vessel disease

Hidehiro Ishikawa[1*], Atsushi Niwa[1**], Shinya Kato[2], Yuichiro Ii[1], Akihiro Shindo[1], Keita Matsuura[1], Yamato Nishiguchi[1], Asako Tamura[1], Akira Taniguchi[1], Masayuki Maeda[3], Yoshio Hashizume[4], Hidekazu Tomimoto [1**]

Affiliations:

[1] Department of Neurology, Mie University Graduate School of Medicine, 2-174 Edobashi, Tsu, Mie 514-8507, Japan
[2] Radioisotope Facilities for Medical Science, Mie University, Tsu, Mie 514-8507, Japan
[3] Department of Advanced Diagnostic Imaging, Mie University Graduate School of Medicine, 2-174 Edobashi, Tsu, Mie 514-8507, Japan
[4] Department of Neuropathology, Fukushimura Hospital, Aichi 441-8124, Japan

*first author

**co-senior author

Corresponding Author:

Dr. Hidehiro Ishikawa
Mie University Graduate School of Medicine, Department of Neurology
2-174 Edobashi Tsu city, Mie 514-8507, JAPAN
E-mail: hidehiro-i@clin.medic.mie-u.ac.jp

SUPPLEMENTARY METHODS

Case presentation

Case 1

A 77-year-old male patient with a 2-year history of progressive memory loss was diagnosed with Alzheimer's disease (AD). His Mini-Mental State Examination score (MMSE) was 11. At age 80, he could not eat due to the progression of AD. A 1.5T-MRI revealed hippocampal atrophy on T1 weighted imaging (WI) and WMH of Fazekas grade III on T2WI (Supplementary Figure 1A, B). On T2*WI, multiple CMBs and cSS were detected, which were compatible with probable CAA according to the modified Boston criteria (Supplementary Figure 1C, D).[1] Nine months after MRI study, he died of pneumonia and autopsy was performed. His neuropathological examination showed neuritic plaque score C on Consortium to Establish a Registry for Alzheimer's Disease (CERAD), stage VI on Braak classification for neurofibrillary tangles (NFT) and severe CAA (Supplementary Figure 2A, B and C).[2,3]

Case 2

A 77-year-old male patient with 1-year history of progressive muscle weakness and atrophy was diagnosed with clinically probable laboratory supported amyotrophic lateral sclerosis (ALS).[4] On admission, his main complaint was difficulty in swallowing and breathing, with % vital capacity (VC) of 58.7%. His MMSE was 29. He was diagnosed with atrial fibrillation (AF) at 73 years of age, and was taking warfarin medication as a result. His brain MRI showed WMH of Fazekas grade I and CMBs in subcortical white matter on susceptibility weighted imaging (SWI) (Supplementary Figure 3A, B). Although his dysphagia was severe and respiratory dysfunction worsened (% VC of 49%) 2 weeks after admission, he and his family refused to use gastrostomy or ventilators. He was transferred to supportive care and died 49 days after MRI scan due to respiratory failure. His neuropathological examination showed pallor and gliosis in the pyramidal tract and a loss of spinal cord horn neurons.

Case 3

A 61-year-old male patient. He presented dementia with MMSE score of 13. He was clinically diagnosed with corticobasal degeneration. His MRI revealed atrophy of right cerebral hemisphere and a CMB in left white matter. One year later, he died of aspiration pneumonia.

Case 4

A 76-year-old male patient with history of arterial fibrillation presented with acute hemiparesis and was taken to the hospital by ambulance. There was a large cerebral hyperintensity in the left middle-cerebral artery area, small hyperintensities in right middle and posterior cerebral artery area, and a micro-hyperintensity in the left occipital lobe compatible with an acute CMI as defined by MRI. He was diagnosed with cardiogenic cerebral embolism. He died of respiratory failure due to severe pneumonia 17 days after the MRI.

Supplementary Figure 1. Neuroradiology for Case 1.

The images shown in Panel A-D was acquired during the patient's hospitalization. A coronal T1-weighted image revealed atrophy of bilateral hippocampus (A). An axial T2-weighted fluid-attenuated inversion recovery (FLAIR) image showed white matter hyper-intensity (B). An axial T2*-weighted image showed a cerebral microbleed (C; arrow) and cortical superficial siderosis (D; arrows).

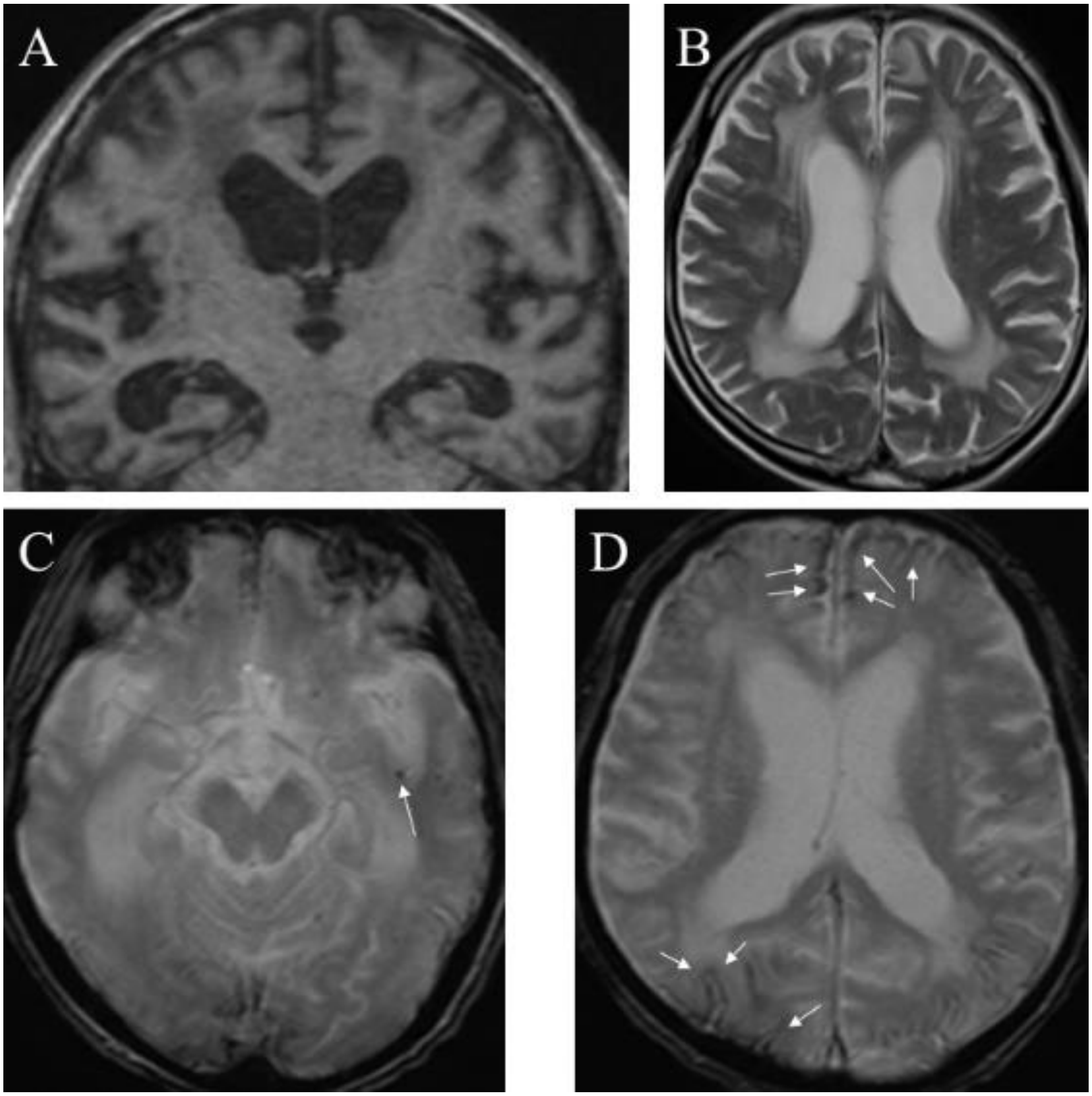

Supplementary Figure 2. Neuropathology for Case 1.

Neuritic plaque (A) and neurofibrillary tangles (B) were observed on Gallyas-Braak staining. Amyloid positive vessels were observed on amyloid staining (C).

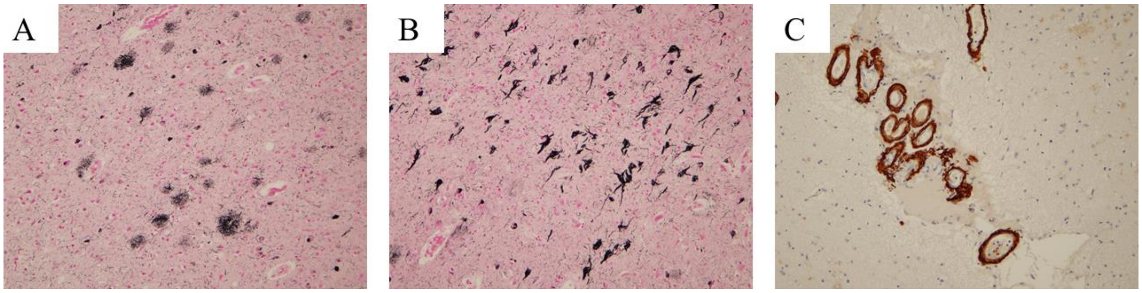

Supplementary Figure 3. Neuroradiology for Case 2

Hypointensity in cortex (A) and white matter (B) were observed on susceptibility weighted image.

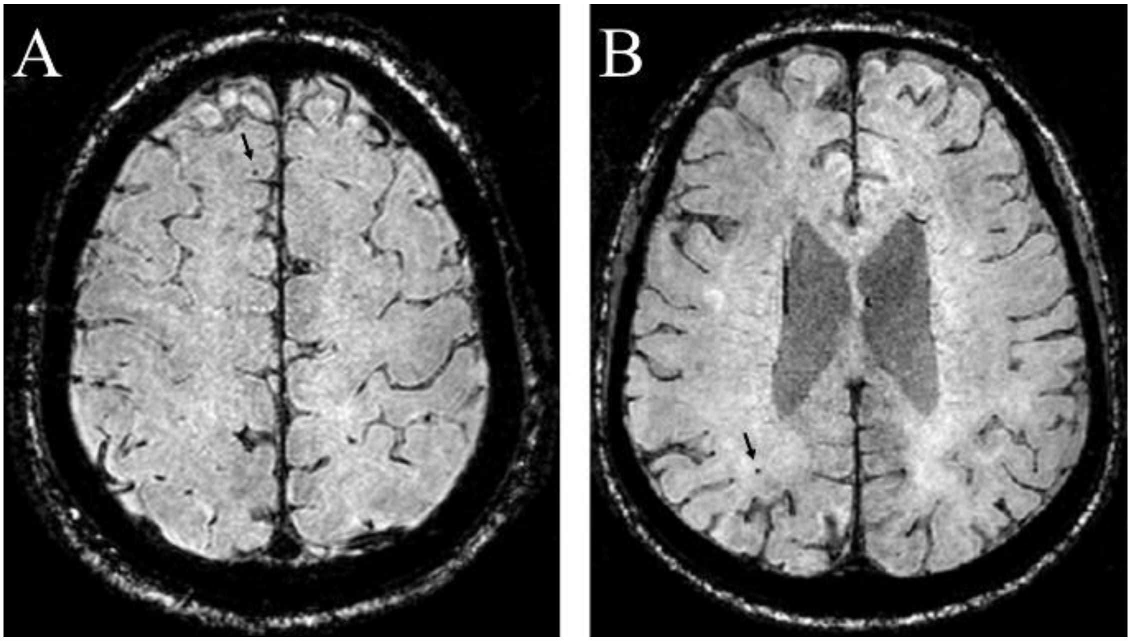

Supplementary Figure 4. Immunohistochemistry of foamy structures in Case 1.
Double staining with iron and additional antibodies. CD68 was negative (A). Ubiquitin was slightly positive or equivocal (B). Although activated astrocytes were gathered around the components, GFAP was negative for the foamy structures (C). MSR (D), NF-L (E) and SMI31(F) were also negative. (Bar=50 µm)

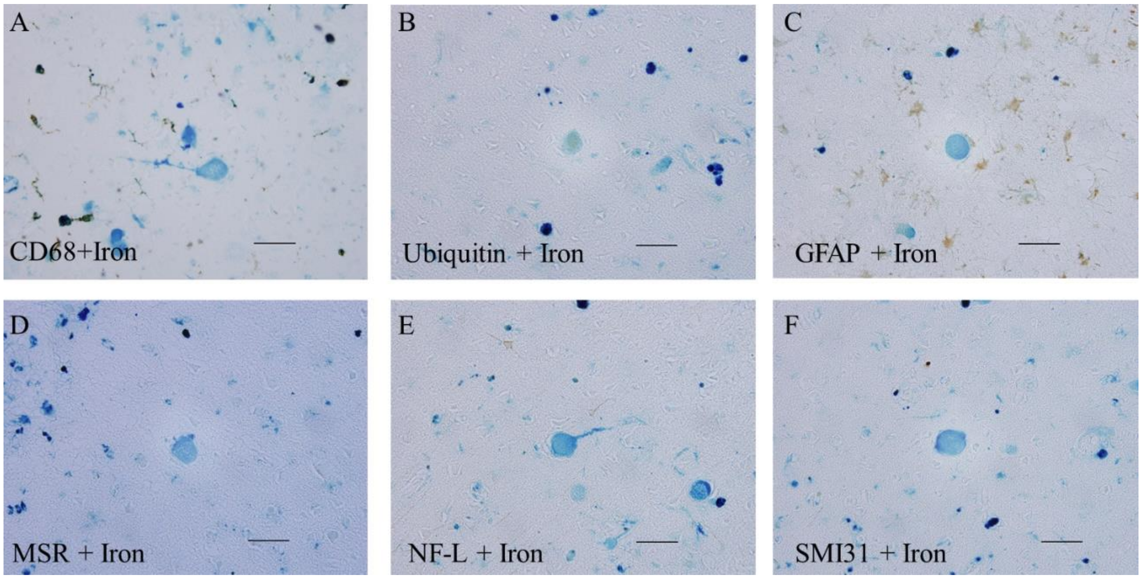

Supplementary Figure 5. Comparative findings of superficial siderosis in Case 2.

There were no findings of cortical superficial siderosis by *in vivo* MRI (A). Hypointensity area along with the surface of cortex was observed by *ex vivo* micro MRI (B). The iron positive area detected by neuropathological iron staining matched the hypointensity area (C; Bar=1 mm and inset of B). Iron-positive cells were of monocyte/macrophage lineages and anuclear foamy structures were not observed in Case 2 (D; Bar=100 μm). There were no Aβ positive vessels in sections of this case on Aβ staining (E; Bar=1 mm).

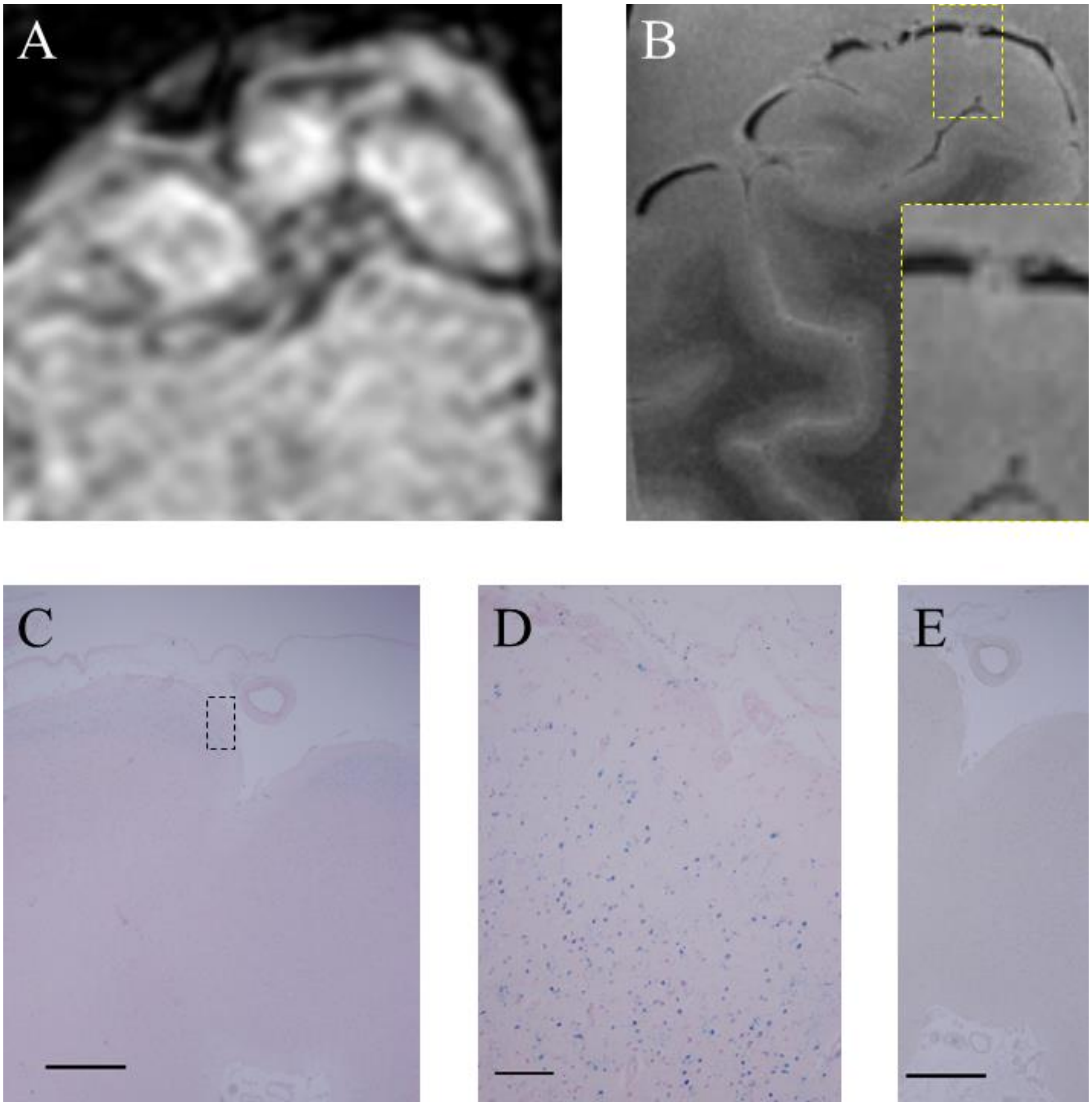

Supplementary Figure 6. Pathological findings of venous angioma in Case 3

The angioma was detected on hematoxylin/eosin (HE) staining. On iron staining, iron positive area was revealed around the angioma.

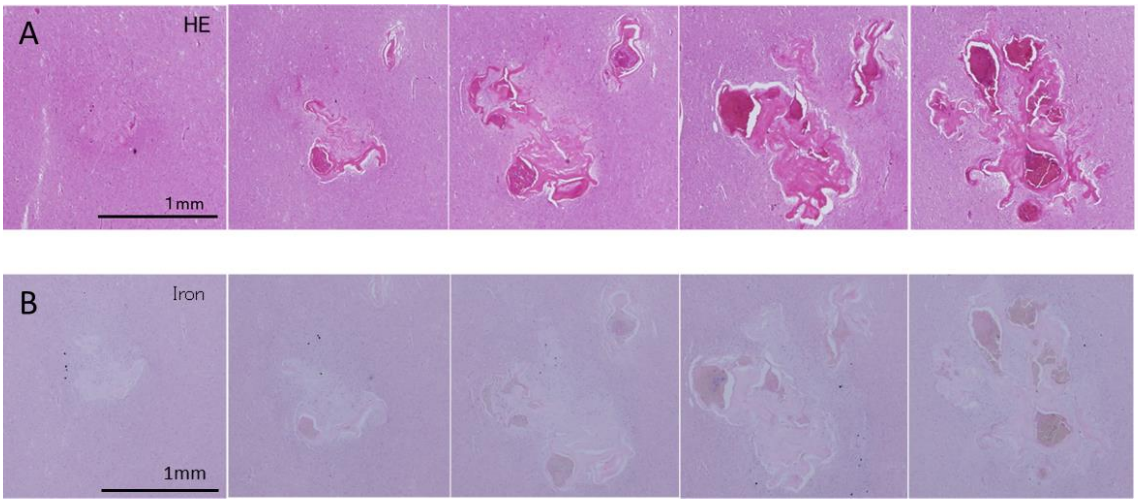

Supplementary Table 1. Antibodies used in immunohistochemistry

Antigen	Catalog number	Antibody type	Dilution	Source
Aβ40	44348A	Rabbit	1000	invitrogen
Aβ42	44344	Rabbit	400	invitrogen
ApoE	ab32897	Goat	400	Abcam
C3d	A0063	Rabbit	200	DAKO
CD31	ab9498	Mouse	100	Abcam
CD68	ab31630	Mouse	100	Abcam
CD204	Ab123946	Rabbit	200	Abcam
Ferritin	Ab76768	Rabbit	100	Abcam
Fibrinogen	GTX26666	Goat	500	Gene Tex
GFAP	MAB360	Mouse	500	MILLIPORE
MSR	AF2708	Goat	500	R&D System
NF-L	C28E10	Rabbit	100	Cell Signaling
PRX1	Ab41906	Rabbit	1000	Abcam
SMI31	801601	Mouse	2000	BioLegend
Transferrin	A0061	Rabbit	1000	DAKO
Ubiquitin	10201-2-AP	Rabbit	200	Proteintech

Supplementary references

1. Greenberg SM, Charidimou A. Diagnosis of Cerebral Amyloid Angiopathy: Evolution of the Boston Criteria. *Stroke* 2018;49:491-497.
2. Mirra SS, Heyman A, McKeel D, et al. The Consortium to Establish a Registry for Alzheimer's Disease (CERAD). Part II. Standardization of the neuropathologic assessment of Alzheimer's disease. *Neurology* 1991;41:479-486.
3. Braak H, Braak E. Neuropathological stageing of Alzheimer-related changes. *Acta Neuropathol* 1991;82:239-259.
4. Brooks BR, Miller RG, Swash M, Munsat TL. El Escorial revisited: revised criteria for the diagnosis of amyotrophic lateral sclerosis. Amyotrophic lateral sclerosis and other motor neuron disorders: official publication of the World Federation of Neurology, Research Group on Motor Neuron Diseases 2000;1:293-299.
